# An optimized rapid bisulfite conversion method with high recovery of cell-free DNA

**DOI:** 10.1186/s12867-017-0101-4

**Published:** 2017-12-19

**Authors:** Shaohua Yi, Fei Long, Juanbo Cheng, Daixin Huang

**Affiliations:** Department of Forensic medicine, Tongji Medical College, Huazhong University of Science and Technology, 13 Hangkong Rd, Wuhan, 430030 China

**Keywords:** Bisulfite conversion, Rapid deamination, High recovery, Cell-free DNA, Methylation analysis, droplet digital PCR

## Abstract

**Background:**

Methylation analysis of cell-free DNA is a encouraging tool for tumor diagnosis, monitoring and prognosis. Sensitivity of methylation analysis is a very important matter due to the tiny amounts of cell-free DNA available in plasma. Most current methods of DNA methylation analysis are based on the difference of bisulfite-mediated deamination of cytosine between cytosine and 5-methylcytosine. However, the recovery of bisulfite-converted DNA based on current methods is very poor for the methylation analysis of cell-free DNA.

**Results:**

We optimized a rapid method for the crucial steps of bisulfite conversion with high recovery of cell-free DNA. A rapid deamination step and alkaline desulfonation was combined with the purification of DNA on a silica column. The conversion efficiency and recovery of bisulfite-treated DNA was investigated by the droplet digital PCR. The optimization of the reaction results in complete cytosine conversion in 30 min at 70 °C and about 65% of recovery of bisulfite-treated cell-free DNA, which is higher than current methods.

**Conclusions:**

The method allows high recovery from low levels of bisulfite-treated cell-free DNA, enhancing the analysis sensitivity of methylation detection from cell-free DNA.

**Electronic supplementary material:**

The online version of this article (10.1186/s12867-017-0101-4) contains supplementary material, which is available to authorized users.

## Background

Analyzing methylation of cell-free DNA (cfDNA) in plasma and other body fluids is a encouraging tool for cancer diagnosis, monitoring and prognosis. Due to the very small amounts of cfDNA in body fluids of normal people and cancer patients [1–4], analytical sensitivity is a very important matter. Most of current methods for analyzing DNA methylation are based on bisulfite-mediated deamination of cytosine [5–8]. During bisulfite treatment, cytosine is quickly converted to uracil, whereas 5-methylcytosine is only slowly changed to thymine.

An important precondition for the usefulness of cfDNA as a diagnostic or prognostic marker for cancer is to easily analyze a small amount of converted DNA. However, the recovery of bisulfite-converted DNA is usually very poor to downstream analysis due to DNA degradation caused by sodium bisulfite treatment [9–11]. The genomic DNA (gDNA) degradation caused by bisulfite treatment results in DNA fragments of an average length of approximately six hundred bases [12]. The chain breakage of cfDNA after bisulfite treatment produces smaller size than its original average 180 bp size [13] and usually limits the following detection step. Fragmentation affects recovery of cfDNA seriously after bisulfite treatment. If the starting amount of DNA is small, most of bisulfite-treated DNA is lost during purification with standard procedures [9, 12]. This is a serious challenge for the analyses of cfDNA with very small amounts of DNA available.

Several published improvements of bisulfite-conversion include a fast deamination step, reducing incubation time from 12 to 16 h to 40 min, succeeded by using a highly concentrated bisulfite solution at higher temperatures [14, 15]. The stepped-up method leads to a more homogeneous conversion of cytosine in a very short time due to the easier process of DNA denaturation in concentrated bisulfite solution at high temperature [16]. Several reliable methods have been put into optimization of the detection of minor amounts of bisulfite-converted gDNA [7, 17, 18]. The PCR technology seems to be a suitable approach generally accepted in the scientific community. It allows for high sensitivity and quantification by droplet digital PCR (ddPCR) [19–22]. However, only one study have addressed the loss of analytic sensitivity associated with recovery of fragmented cfDNA caused by the bisulfite treatment [23]. Here we reported a optimized rapid method with a higher recovery of bisulfite-treated cfDNA for the detection of methylation.

## Materials and methods

### cfDNA samples

Plasma samples were collected from the patients of liver cancer and breast cancer. The procedures were approved by the Ethics Committee of Human Experimentation in our college. cfDNA was isolated from 3 mL plasma on Hi-pure Circulating DNA Midi Kit (Magen) using the recommended protocol.

### Bisulfite treatment

Bisulfite treatment was based on previously published accelerated methods [14, 15] with some modifications: 130 μL of 10 M (NH4) HSO3–NaHSO3 bisulfite solution was added to 20 μL cfDNA in PCR tubes. The mixtures were heated for 30 min at 70 °C or for 10 min at 90 °C and subsequently cooled to 4 °C in a PCR machine. The bisulfite treated DNA solution was purified with the Zymo-Spin IC Columns (Zymo) according to manufacture’s instructions with the change: DNA was eluted with 20 μL Elution Buffer. On the other hand, In order to compare the conversion efficiency of the above method with that of a commercial protocol, the EZ DNA Methylation-Lightning Kit (Zymo) was used to convert the parallel cfDNA samples as the manufacturer’s protocol.

### DNA quantification

**Fig. 1:**
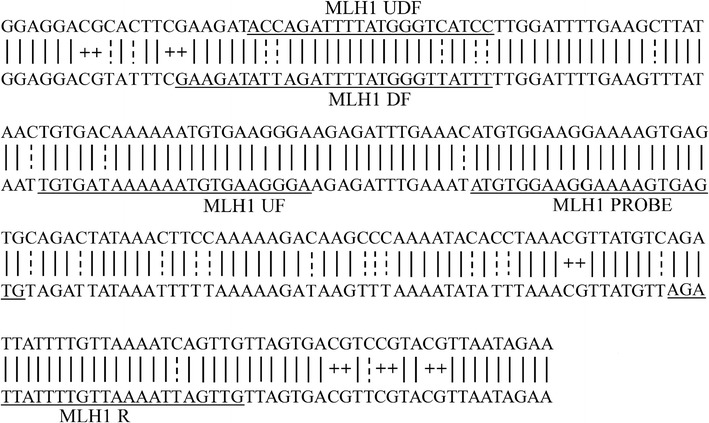
Binding sites of primers and probe used are underlined [23]. The top strand is the undeaminated DNA sequence. Bottom strand is the deaminated sequence. A solid line between the two strands illustrates no difference in sequence, a dotted line marks the positions of cytosine converted to uracil during deamination, and “+” marks the positions of CpG dinucleotides. Primer sequences can be found in Table 1. The universal primers, MLH1 UF and MLH1 R, contain Inosine at one position each

The conversion efficiency and recovery of bisulfite-treated DNA was investigated by absolute quantification using the ddPCR technique. To investigate the recovery of bisulfite-treated DNA, three different primer sets sharing the same detection probe were designed as the published method [23]: MLH1 UF and MLH1 R detected DNA regardless of deamination. MLH1 DF and MLH1 R detected deaminated DNA, whereas MLH1 UDF and MLH1 R detected undeaminated DNA (Table 1 and Fig. 1). For the optimization of the ddPCR conditions, 52–58 °C turned out to be an optimal temperature for all assays and was used for further analyses. For absolute quantification, the QX200 Droplet Digital PCR system (Bio-Rad) was used. 5 μL of template DNA was mixed in a 20 μL reaction volume with 10 μL 2 × ddPCR Supermix for Probes (No dUTP) (Bio-Rad), 2 μL of the primers, 1 μL probe mix and 2 μL DNase-free water. Samples were mixed with Droplet Generation Oil for Probes (Bio-Rad) according to the manufacturer’s instructions and droplets generated in a QX200 Droplet Generator (Bio-Rad). PCR conditions were 95 °C for 10 min, 40 cycles of 94 °C for 30 s and 52–58 °C for 1 min, and 98 °C for 10 min. Droplets were read on the QX200 Droplet Reader (Bio-Rad) and data analyzed with QuantaSoft Software. Analyses were done in triplicates and Negative water controls were always included.

**Table 1:** Primer and probe sequences

Primer/probe	Sequence (5′–3′)
MLH1 UF	TGTGAIAAAAAATGTGAAGGG
MLH1 DF	GAAGATATTAGATTTTATGGGTTATTT
MLH1 R	CAACTIATTTTAACAAAATAATCT
MLH1 UDF	ACCAGATTTTATGGGTCATCC
MLH1 PROBE	(FAM)CGCGAATGTGGAAGGAAAAGTGAGTGTCGC(TAMRA)
MTND4P12 UF	TAGTAGGTTAATAGTGGGG
MTND4P12 R	ACTTACATCCTCATTACTATTCT
MTND4P12 PROBE	(FAM)CGCGATTAGTGGGAGTAGGGTTTGAAGTCGC(TAMRA)
RASSF1A UF	GTTTTGGTAGTTTAATGAGTTTAGGTT
RASSF1A R	CCCCACAATCCCTACACCCAAAT
RASSF1A PROBE	(FAM)CGCGATGGATYYTGGGGGAGGTCGCG(TAMRA)

MLH1 UF and MLH1 R concurrently amplify total deaminated and undeaminated MLH1 promoter. MLH1 DF and MLH1 R amplify deaminated MLH1. MLH1 UDF and MLH1 R amplify undeaminated MLH1 promoter. A common MLH1 probe was used for detection of the three products. MTND4P12 UF and MTND4P12 R amplify the MTND4P12 sequence regardless of the deamination. RASSF1A UF and RASSF1A R amplify total deaminated and undeaminated RASSF1A sequence

### Measurement of deamination efficiency

The plasma cfDNA from twenty patients of breast cancer was extracted to measure and evaluate the deamination efficiency of the optimized conversion method. To prove the finding a universal approach, we investigated whether the methylation status of the unmethylated CpG islands of MLH1 and MTND4P12 and the hyper-methylated CpG islands of RASSF1A in cancers [24] can be reproduced by our method in plasma from serious breast cancer patients. The primers of MTND4P12 and ASSF1A were shown in Table 1. The PCR product was purified and cloned into pUC19 plasmid (Takara Bio). Fifteen independent plasmid clones were picked up and subjected to sequence analysis.

### Measurement of DNA recovery

The plasma cfDNA from twenty patients of liver cancer was extracted to measure the recovery of converted DNA. 20 μL cfDNA was deaminated and purified, another 20 μL cfDNA was mock-deaminated (treated only with TE) and purified, whereas the residual used for quantitation using MLH1 UF and R primers described in Table 1. The recovery of converted cfDNA from our optimized method and the EZ DNA methylation-lightning kit was also measured using MTND4P12 and RASSF1A primers (Table 1).

## Results

**Fig. 2:**
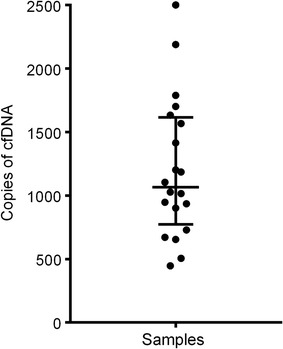
Copies of cell-free DNA detected by ddPCR. The copy number represents the total amount of copies per milliliter plasma samples

The optimization of ddPCR reaction conditions resulted in a well-performing assay (Additional file 1). Excellent separation between positive droplets and negative droplets contributed to an accurate assay for the determination of the concentration of the target DNA molecule. The detected copies were calculated as the amount of copies per milliliter plasma. The copies of cell-free DNA vary largely from different samples (Fig. 2).

**Fig. 3:**
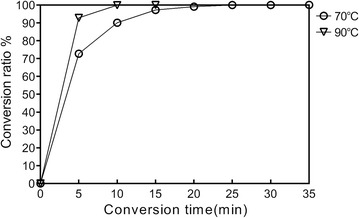
The analysis of the efficiency of the conversion. Open circles indicate the ratio of conversion of unmethylated cytosine to uracil at 70 °C. Open triangles indicate the ratio of conversion of unmethylated cytosine to uracil at 90 °C

**Fig. 4:**
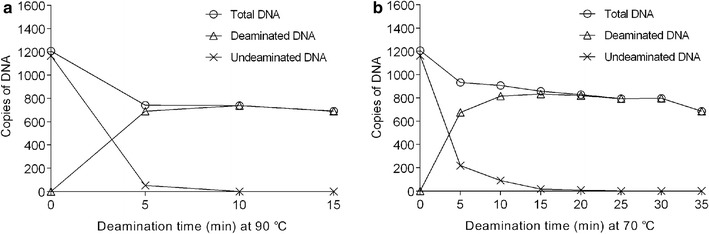
**a** Dynamics monitoring of the deamination reaction at 90 °C. **b** Dynamics monitoring of the deamination reaction at 70 °C. Monitoring of the deamination reaction as a function of deamination time using primers designed for an unmethylated part of the MLH1 promoter. Three different primer sets were used to amplify total deaminated and undeaminated DNA, deaminated DNA, and undeaminated DNA separately. ddPCR detection of the three products was performed with a common molecular probe. The copy number represents the average amount of copies from 20 plasma samples. Open circles indicate the copies of total DNA. Open triangles indicate the copies of deaminated DNA. Asterisks mean the copies of undeaminatd DNA

Dynamics of the reaction have been extensively monitored using a ddPCR based method. The results of optimization of the reaction time are shown in Figs. 3 and 4. When the DNA was treated at 90 °C for a 5-min incubation, about 93% of the cytosine residues in the analyzed region were converted to uracil (Fig. 3). After a 10-min incubation, more than 99.5% of cytosine residues were converted to uracil (Fig. 3). A 15-min incubation resulted in almost 100% conversion while the detected copies of deaminated DNA and total DNA began to decline (Figs. 3, 4a). These results demonstrate that a complete conversion of cytosine to uracil with the biggest recovery of DNA can be achieved at 90 °C within 10 min.

When the DNA was treated at 70 °C, a reduced rate of conversion was observed (Figs. 3, 4b). After a 5 min of deamination, about 73% of the cytosine residues in the analyzed region were converted to uracil (Fig. 3). After a 25-min incubation, more than 99.5% of cytosine residues were converted to uracil (Fig. 3) while a 30-min incubation resulted in almost 100% conversion (Fig. 3). After a longer time of incubation, the detected copies of deaminated DNA and total DNA began to decline (Fig. 4b). Therefore, a 30-min incubation at 70 °C can result in complete conversion of cytosine to uracil with the biggest recovery of DNA.

**Fig. 5:**
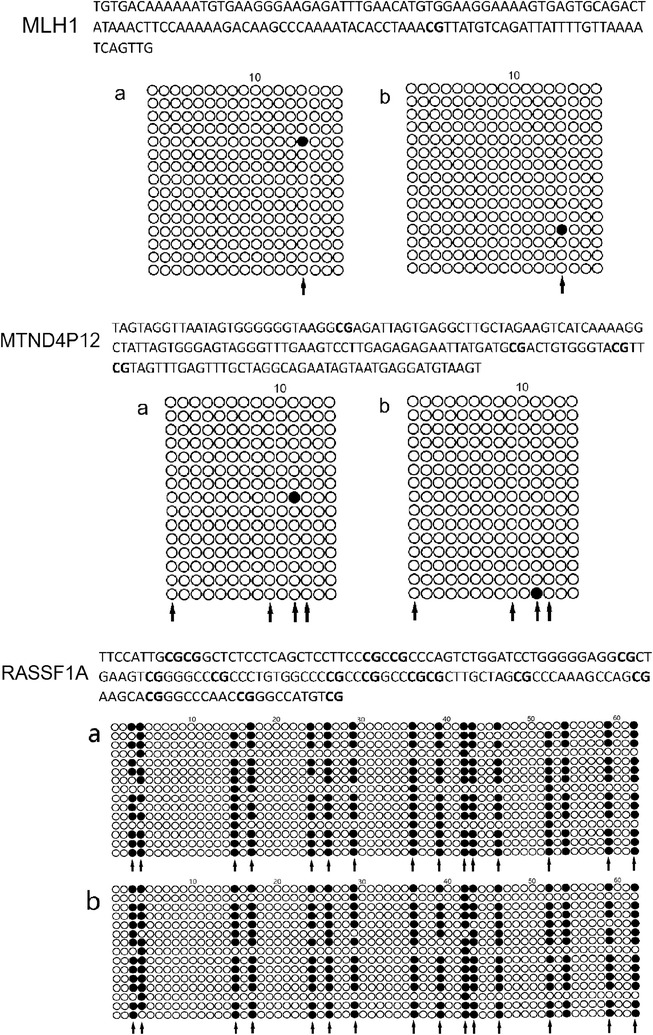
Bisulfite genomic sequencing analysis of three genes in plasma cfDNA of breast cancers. In each nucleotide sequence of the region, bold characters indicate CpG dinucleotides. For nucleotide sequence of the RASSF1A region, The complementary strand was used as a template. As a consequence, cytosine methylation status of the complementary strand is reflected as guanine residues. Nucleotide sequence analysis of plasmid clones: **a** treatment with Zymo EZ DNA methylation-lightening kit; **b** 70 °C for 30 min. Each row indicates an independent plasmid clone. Open and closed circles indicate cytosines and methylcytosines, respectively. Arrows indicate positions of cytosine at CpG dinucleotides

For the bisulfite sequencing results (Additional file 2), when the plasma DNA was treated by the Zymo EZ DNA methylation-lightening kit, all cytosine residues at non-CpG sites in three genes were converted to uracil in all 15 plasmid clones that were analyzed (Fig. 5a). Almost the same results were obtained when the same sample was treated by our optimized method for 30 min at 70 °C (Fig. 5b). We then analyzed the methylation status of the cytosine residues at CpG sites of three genes. Most cytosine residues at CpG sites of MLH1 and MTND4P12 were modified (Fig. 5). In contrast, most cytosine residues at CpG sites of RASSF1A remained unmodified (Fig. 5). Almost the same results were obtained when the same sample was treated with our method for 30 min at 70 °C (Fig. 5).

**Fig. 6:**
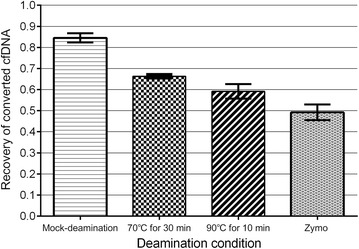
Recovery of bisulfite-treated cfDNA based on four deamination conditions: mock-deamination, deamination at 70 °C for 30 min and deamination at 90 °C for 10 min, deamination using Zymo EZ methylation lightening Kit. Recovery means the average recovery of twenty individual deamination reactions. Significant differences of recovery of bis-cfDNA exist among these methods, *P* < 0.0001

DNA is damaged at pyrimidine sites during reaction with bisulfite, as described above. The amount of target degradation during bisulfite incubation was quantified with ddPCR (Fig. 6, Table 2). For the MLH1 PCR products, the average recovery of cfDNA treated by Zymo EZ DNA methylation-lightening kit decreased to 50.3%. After bisulfite treatment for 10 min at 90 °C, the average recovery of 20 samples decreased to approximately 59.2%. After bisulfite treatment for 30 min at 70 °C, 66.3% of converted cfDNA was recovered. The average recovery of cfDNA was still up to 84.5% after a mock conversion only with TE buffer treatment. For the PCR products of MLH1, RASSF1A and MTND4P12, the average recovery of cfDNA is about 65%, still higher than that of the commercial kits (Table 2).

**Table 2:** Recovery of bisulfite-treated cfDNA (n = 20)

Conversion method	MLH1	RASSF1A	MTND4P12
Deamination at 70 °C for 30 min	0.663 ± 0.009	0.641 ± 0.037	0.650 ± 0.039
Deamination by Zymo kit	0.503 ± 0.037	0.456 ± 0.061	0.497 ± 0.040
*P* value	< 0.0001	< 0.0001	< 0.0001

## Discussion

A previously published bisulfite treatment method has led to the development of a fast bisulfite conversion of DNA [15]. Optimization involved the fast treatment in a highly concentrated bisulfite solution at higher temperatures and combined purification procedure after deamination, which contribute to the recovery of the fragmented DNA resulting from bisulfite treatment [12, 23]. The dynamics of the reaction were measured to monitor optimal conversion of cytosine and limit conversion of methyl-cytosine.

It is of utmost importance for sequencing individual clones to completely deaminate the unmethylated cytosine. A false positive result could be obtained from incomplete deamination of unmethylated cytosine. Complete conversion of cytosine could be achieved with only a limited amount of cytosines (< 1%) was detectable after 10 min deamination at 90 °C or 20 min deamination at 70 °C [15]. Smaller amount of both deaminated and undeaminated DNA could be detected after longer deamination time [15]. DNA were less severely damaged when treated with 10 M bisulfite at 70 °C compared to 90 °C. Serious degradation of template DNA happened when samples were treated with the buffer for more than 10 min deamination at 90 °C or 40 min deamination at 70 °C [15]. Therefore, optimization of the reaction time had a pronounced effect on recovery. Since the purpose of this procedure is to optimize this protocol in order to achieve the best possible recovery of bisulfite treated DNA, we compared the recovery of deaminated cfDNA at two different deamination condition.

After a 10-min deamination at 90 °C, no undeaminated product was detectable by ddPCR (Fig. 4a). A complete conversion of cytosine to uracil and a 59.2% of cfDNA was achieved within 10-min conversion. Longer deamination time than 10 min results in reduced detection of both deaminated and undeaminated DNA. When the cfDNA was treated at 70 °C for 30 min, a reduced rate of degradation was observed (Fig. 4b) and 66.3% of cfDNA was recovered with complete conversion of cytosine to uracil. Therefore, 30-min deamination at 70 °C could achieve higher recovery of bisulfite-converted cfDNA than 10-min deamination at 90 °C. Deamination at 70 °C resulted in a lower speed of degradation of DNA than deamination at 90 °C.

The ddPCR assay capable of detecting both treated and untreated DNA allows the same assay to be used for measuring DNA levels both pre- and post- bisulfite treatment resulting in improved accuracy [11]. The probe and primers used in this study have been designed in order to be specific for either the methylated or the unmethylated sequence and give parallel reaction conditions. No cross-reactivity is observed in our experiment. Inappropriate conversion of methylated cytosine could lead to reduced sensitivity when detecting methylated DNA. The sequencing data shows that no over-conversion is detected by the ddPCR based experiment because the primers and the probe are designed to be specific for an unmethylated part of MLH1.

In order to prove the method a universal approach, three genes with different sequence features were amplified to measure the conversion and recovery efficiency of the cfDNA. The sequencing data of their PCR products demonstrates that all the unmethylated cytosine could be modified and no change of methylated cytosine were achieved after 30 min deamination at 70 °C. The fast method could get the same conversion efficiency as the commercial methylation kit.

A conventional bisulfite modification usually needs denaturation of DNA prior to bisulfite treatment [13]. No denaturants in the protocol presented here possibly reflect DNA denaturation and bisulfite conversion processes are combined and the high concentration of bisulfite and high temperature facilitate rapid denaturation. Desulphonation and cleanup of the converted DNA are performed using a unique low-elution spin column. Re-extraction of cfDNA in this matrix results in a recovery of 84.5% whereas average recovery of deaminated DNA is 65% (Table 2). The recovery of deaminated cfDNA is more considerable compared with that of the Zymo kit and other published methods [20]. This work addresses the problem of poor recovery and contribute to the methylation detection of the minor amount of cfDNA. The lower deamination temperature facilitates the higher rate of intact bisulfite-treatment cfDNA and the use of purification kits helps recover most of the denatured cfDNA fragments.

## Conclusions

Our work optimized a fast-speed and encouraging bisulfite treatment with high recovery from cfDNA samples, improving analytical sensitivity of the potential methylation markers from body fluid.

## Additional files

**Additional file 1: Figure.** An example of a ddPCR assay for absolute quantification of DNA copies. The designed ddPCR reaction produced an excellent separation between positive droplets(top) and negative droplets(bottom). The amplitude threshold of the positive ddPCR reaction was set as 4000 RFU manually. The positive droplets above the threshold line determines the starting concentration of the target DNA molecule in units of copies/µL input from the sample.

**Additional file 2: Data.** Bisulfite sequencing and DNA methylation data.

## Abbreviations

cfDNA: cell-free DNA
gDNA: genomic DNA
ddPCR: droplet digital PCR
MLH1UF: forward primer of MLH1 detecting DNA regardless of deamination
MLH1 R: reverse primer of MLH1 detecting DNA regardless of deamination
MLH1 DF: forward primer of MLH1 detecting deaminated DNA
MLH1 UDF: forward primer of MLH1 detecting undeaminated DNA
MTND4P12 UF: forward primer of MTND4P12 detecting DNA regardless of deamination
MTND4P12 R: reverse primer of MTND4P12 detecting DNA regardless of deamination
RASSF1A UF: forward primer of RASSF1A detecting DNA regardless of deamination
RASSF1A R: forward primer of RASSF1A detecting DNA regardless of deamination

## References

[1] Yoon KA, Park S, Lee SH, Kim JH, Lee JS (2009). Comparison of circulating plasma DNA levels between lung cancer patients and healthy controls. J Mol Diagn..

[2] Jung K, Fleischhacker M, Rabien A (2010). Cell-free DNA in the blood as a solid tumor biomarker—a critical appraisal of the literature. Clin Chim Acta.

[3] Szpechcinski A, Chorostowska-Wynimko J, Struniawski R, Kupis W, Rudzinski P, Langfort R (2015). Cell-free DNA levels in plasma of patients with non-small-cell lung cancer and inflammatory lung disease. Br J Cancer.

[4] Tissot C, Toffart AC, Villar S, Souquet PJ, Merle P, Moro-Sibilot D (2015). Circulating free DNA concentration is an independent prognostic biomarker in lung cancer. Eur Respir J.

[5] Oakes CC, La Salle S, Robaire B, Trasler JM (2006). Evaluation of a quantitative DNA methylation analysis technique using methylation-sensitive/dependent restriction enzymes and real-time PCR. Epigenetics.

[6] Zhang Y, Rohde C, Tierling S, Stamerjohanns H, Reinhardt R, Walter J (2009). DNA methylation analysis by bisulfite conversion, cloning, and sequencing of individual clones. Methods Mol Biol.

[7] Paliwal A, Vaissiere T, Herceg Z (2010). Quantitative detection of DNA methylation states in minute amounts of DNA from body fluids. Methods.

[8] Wang T, Guan W, Lin J, Boutaoui N, Canino G, Luo J (2015). A systematic study of normalization methods for Infinium 450 K methylation data using whole-genome bisulfite sequencing data. Epigenetics.

[9] Tanaka K, Okamoto A (2007). Degradation of DNA by bisulfite treatment. Bioorg Med Chem Lett.

[10] Grunau C, Clark SJ, Rosenthal A (2001). Bisulfite genomic sequencing: systematic investigation of critical experimental parameters. Nucleic Acids Res.

[11] Mill J, Petronis A (2009). Profiling DNA methylation from small amounts of genomic DNA starting material: efficient sodium bisulfite conversion and subsequent whole-genome amplification. Methods Mol Biol.

[12] Munson K, Clark J, Lamparska-Kupsik K, Smith SS (2007). Recovery of bisulfite-converted genomic sequences in the methylation-sensitive ddPCR. Nucleic Acids Res.

[13] Jahr S, Hentze H, Englisch S, Hardt D, Fackelmayer FO, Hesch RD (2001). DNA fragments in the blood plasma of cancer patients: quantitations and evidence for their origin from apoptotic and necrotic cells. Cancer Res.

[14] Hayatsu H, Negishi K, Shiraishi M (2004). DNA methylation analysis: speedup of bisulfate-mediated deamination of cytosine in the genomic sequencing procedure. Proc Jpn Acad Ser B..

[15] Shiraishi M, Hayatsu H (2004). High-speed conversion of cytosine to uracil in bisulfite genomic sequencing analysis of DNA methylation. DNA Res.

[16] Genereux DP, Johnson WC, Burden AF, Stoger R, Laird CD (2008). Errors in the bisulfite conversion of DNA: modulating inappropriate and failed conversion frequencies. Nucleic Acids Res.

[17] Vaissière T, Cuenin C, Paliwal A, Vineis P, Hoek G, Krzyzanowski M (2009). Quantitative analysis of DNA methylation after whole bisulfitome amplification of a minute amount of DNA from body fluids. Epigenetics.

[18] Rajput SK, Kumar S, Dave VP, Rajput A, Pandey HP, Datta TK (2012). An improved method of bisulfite treatment and purification to study precise DNA methylation from as little as 10 pg DNA. Appl Biochem Biotechnol.

[19] Parsons HA, Beaver JA, Park BH (2016). Circulating plasma tumor DNA. Adv Exp Med Biol.

[20] Hudecova I (2015). Digital PCR analysis of circulating nucleic acids. Clin Biochem.

[21] Huggett JF, Cowen S, Foy CA (2015). Considerations for digital PCR as an accurate molecular diagnostic tool. Clin Chem.

[22] Day E, Dear PH, McCaughan F (2013). Digital PCR strategies in the development and analysis of molecular biomarkers for personalized medicine. Methods.

[23] Pedersen IS, Krarup HB, Thorlacius-Ussing O, Madsen PH (2012). High recovery of cell-free methylated DNA based on a rapid bisulfite-treatment protocol. BMC Mol Biol.

[24] Dammann R, Yang G, Pfeifer GP (2001). Hyper-methylation of the CpG island of Ras association domain family 1A (RASSF1A), a putative tumor suppressor gene from the 3p21.3 locus, occurs in a large percentage of human breast cancers. Cancer Res.

