# Supplementary figures and images for: An optimized rapid bisulfite conversion method with high recovery of cell-free DNA
Source: BMC Mol Biol. 2017 Dec 19;18:24. doi: 10.1186/s12867-017-0101-4 (PMC5735811; doi:10.1186/s12867-017-0101-4)

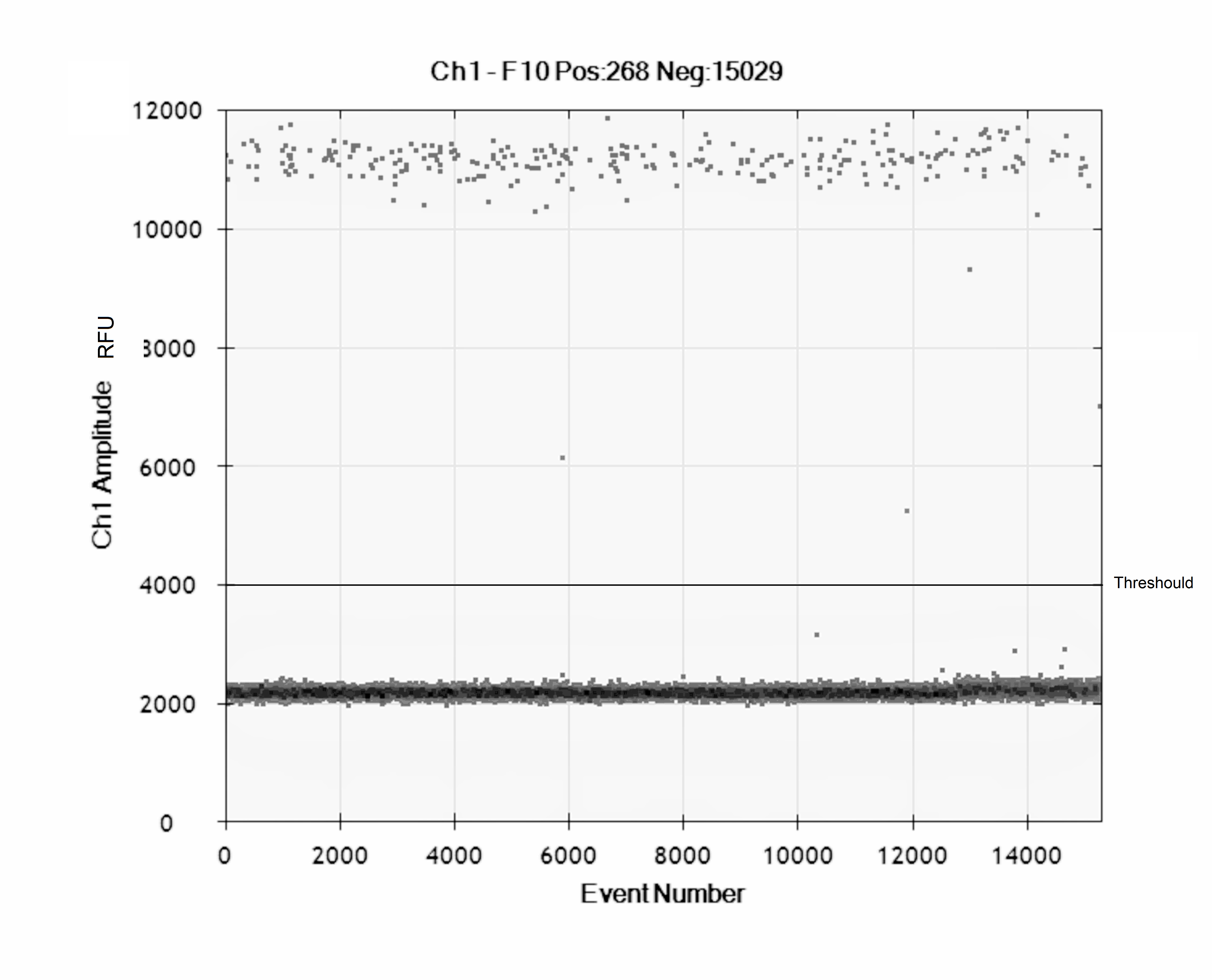

Supplement: Supplementary file 1 — Additional file 1: Figure. An example of a ddPCR assay for absolute quantification of DNA copies. The designed ddPCR reaction produced an excellent separation between positive droplets(top) and negative droplets(bottom). The amplitude threshold of the positive ddPCR reaction was set as 4000 RFU manually. The positive droplets above the threshold line determines the starting concentration of the target DNA molecule in units of copies/µL input from the sample. [file 12867_2017_101_MOESM1_ESM.tif]
