# Supplementary material for: An optimized rapid bisulfite conversion method with high recovery of cell-free DNA
Source: BMC Mol Biol. 2017 Dec 19;18:24. doi: 10.1186/s12867-017-0101-4 (PMC5735811; doi:10.1186/s12867-017-0101-4)
Supplement: Supplementary file 2 — Additional file 2: Data. Bisulfite sequencing and DNA methylation data. [file 12867_2017_101_MOESM2_ESM.pdf]

Additional file 2: Bisulfite sequencing and DNA methylation data

Genename MLH1

Gene Genomic Sequence (original)
TGTGACAAAAAATGTGAAGGGAAGAGATTTGAACATGTGGAAGGAAAAGTGAGTGCAGACTATAAACTTCCAAAAAGACAA
GCCCAAAATACACCTAAA**CG**TTATGTCAGATTATTTTGTTAAAATCAGTTG

a. Bisulfite treatment with Zymo EZ DNA methylation-lightening kit

```
Genomic_Sequence     TGTGACAAAAAATGTGAAGGGAAGAGATTTGAACATGTGGAAGGAAAAGTGAGTGCAGAC
[1]Copy\of\B108-35...TGTGATAAAAAATGTGAAGGGAAGAGATTTGAATATGTGGAAGGAAAAGTGAGTGTAGAT
[2]Copy\of\B108-35...TGTGATAAAAAATGTGAAGGGAAGAGATTTGAATATGTGGAAGGAAAAGTGAGTGTAGAT
[3]Copy\of\B108-35...TGTGATAAAAAATGTGAAGGGAAGAGATTTGAATATGTGGAAGGAAAAGTGAGTGTAGAT
[4]Copy\of\B108-35...TGTGATAAAAAATGTGAAGGGAAGAGATTTGAATATGTGGAAGGAAAAGTGAGTGTAGAT
[5]Copy\of\B108-35...TGTGATAAAAAATGTGAAGGGAAGAGATTTGAATATGTGGAAGGAAAAGTGAGTGTAGAT
[6]Copy\of\B108-35...TGTGATAAAAAATGTGAAGGGAAGAGATTTGAATATGTGGAAGGAAAAGTGAGTGTAGAT
[7]Copy\of\B108-35...TGTGATAAAAAATGTGAAGGGAAGAGATTTGAATATGTGGAAGGAAAAGTGAGTGTAGAT
[8]Copy\of\B108-35...TGTGATAAAAAATGTGAAGGGAAGAGATTTGAATATGTGGAAGGAAAAGTGAGTGTAGAT
[9]Copy\of\B108-35...TGTGATAAAAAATGTGAAGGGAAGAGATTTGAATATGTGGAAGGAAAAGTGAGTGTAGAT
[10]Copy\of\B108-3...TGTGATAAAAAATGTGAAGGGAAGAGATTTGAATATGTGGAAGGAAAAGTGAGTGTAGAT
[11]Copy\of\B108-3...TGTGATAAAAAATGTGAAGGGAAGAGATTTGAATATGTGGAAGGAAAAGTGAGTGTAGAT
[12]Copy\of\B108-3...TGTGATAAAAAATGTGAAGGGAAGAGATTTGAATATGTGGAAGGAAAAGTGAGTGTAGAT
[13]Copy\of\B108-3...TGTGATAAAAAATGTGAAGGGAAGAGATTTGAATATGTGGAAGGAAAAGTGAGTGTAGAT
[14]Copy\of\B108-3...TGTGATAAAAAATGTGAAGGGAAGAGATTTGAATATGTGGAAGGAAAAGTGAGTGTAGAT
[15]Copy\of\B108-3...TGTGATAAAAAATGTGAAGGGAAGAGATTTGAATATGTGGAAGGAAAAGTGAGTGTAGAT

Genomic_Sequence     TATAAACTTCCAAAAAGACAAGCCCAAAATACACCTAAACGTTATGTCAGATTATTTTGT
[1]Copy\of\B108-35...TATAAATTTTTAAAAAGATAAGTTTAAAATATATTTAAATGTTATGTTAGATTATTTTGT
[2]Copy\of\B108-35...TATAAATTTTTAAAAAGATAAGTTTAAAATATATTTAAATGTTATGTTAGATTATTTTGT
[3]Copy\of\B108-35...TATAAATTTTTAAAAAGATAAGTTTAAAATATATTTAAATGTTATGTTAGATTATTTTGT
[4]Copy\of\B108-35...TATAAATTTTTAAAAAGATAAGTTTAAAATATATTTAAATGTTATGTTAGATTATTTTGT
[5]Copy\of\B108-35...TATAAATTTTTAAAAAGATAAGTTTAAAATATATTTAAACGTTATGTTAGATTATTTTGT
[6]Copy\of\B108-35...TATAAATTTTTAAAAAGATAAGTTTAAAATATATTTAAATGTTATGTTAGATTATTTTGT
[7]Copy\of\B108-35...TATAAATTTTTAAAAAGATAAGTTTAAAATATATTTAAATGTTATGTTAGATTATTTTGT
[8]Copy\of\B108-35...TATAAATTTTTAAAAAGATAAGTTTAAAATATATTTAAATGTTATGTTAGATTATTTTGT
[9]Copy\of\B108-35...TATAAATTTTTAAAAAGATAAGTTTAAAATATATTTAAATGTTATGTTAGATTATTTTGT
[10]Copy\of\B108-3...TATAAATTTTTAAAAAGATAAGTTTAAAATATATTTAAATGTTATGTTAGATTATTTTGT
[11]Copy\of\B108-3...TATAAATTTTTAAAAAGATAAGTTTAAAATATATTTAAATGTTATGTTAGATTATTTTGT
[12]Copy\of\B108-3...TATAAATTTTTAAAAAGATAAGTTTAAAATATATTTAAATGTTATGTTAGATTATTTTGT
[13]Copy\of\B108-3...TATAAATTTTTAAAAAGATAAGTTTAAAATATATTTAAATGTTATGTTAGATTATTTTGT
[14]Copy\of\B108-3...TATAAATTTTTAAAAAGATAAGTTTAAAATATATTTAAATGTTATGTTAGATTATTTTGT
[15]Copy\of\B108-3...TATAAATTTTTAAAAAGATAAGTTTAAAATATATTTAAATGTTATGTTAGATTATTTTGT

Genomic_Sequence     TAAAATCAGTTG
[1]Copy\of\B108-35...TAAAATTAGTTG
[2]Copy\of\B108-35...TAAAATTAGTTG
[3]Copy\of\B108-35...TAAAATTAGTTG
[4]Copy\of\B108-35...TAAAATTAGTTG
[5]Copy\of\B108-35...TAAAATTAGTTG
[6]Copy\of\B108-35...TAAAATTAGTTG
[7]Copy\of\B108-35...TAAAATTAGTTG
[8]Copy\of\B108-35...TAAAATTAGTTG
[9]Copy\of\B108-35...TAAAATTAGTTG
[10]Copy\of\B108-3...TAAAATTAGTTG
[11]Copy\of\B108-3...TAAAATTAGTTG
[12]Copy\of\B108-3...TAAAATTAGTTG
[13]Copy\of\B108-3...TAAAATTAGTTG
[14]Copy\of\B108-3...TAAAATTAGTTG
[15]Copy\of\B108-3...TAAAATTAGTTG
```

b. Bisulfite treatment with 70℃ for 30 min

```
Genomic_Sequence     TGTGACAAAAAATGTGAAGGGAAGAGATTTGAACATGTGGAAGGAAAAGTGAGTGCAGAC
[1]Copy\of\B108-35...TGTGATAAAAAATGTGAAGGGAAGAGATTTGAATATGTGGAAGGAAAAGTGAGTGTAGAT
[2]Copy\of\B108-35...TGTGATAAAAAATGTGAAGGGAAGAGATTTGAATATGTGGAAGGAAAAGTGAGTGTAGAT
[3]Copy\of\B108-35...TGTGATAAAAAATGTGAAGGGAAGAGATTTGAATATGTGGAAGGAAAAGTGAGTGTAGAT
[4]Copy\of\B108-35...TGTGATAAAAAATGTGAAGGGAAGAGATTTGAATATGTGGAAGGAAAAGTGAGTGTAGAT
[5]Copy\of\B108-35...TGTGATAAAAAATGTGAAGGGAAGAGATTTGAATATGTGGAAGGAAAAGTGAGTGTAGAT
[6]Copy\of\B108-35...TGTGATAAAAAATGTGAAGGGAAGAGATTTGAATATGTGGAAGGAAAAGTGAGTGTAGAT
[7]Copy\of\B108-35...TGTGATAAAAAATGTGAAGGGAAGAGATTTGAATATGTGGAAGGAAAAGTGAGTGTAGAT
[8]Copy\of\B108-35...TGTGATAAAAAATGTGAAGGGAAGAGATTTGAATATGTGGAAGGAAAAGTGAGTGTAGAT
[9]Copy\of\B108-35...TGTGATAAAAAATGTGAAGGGAAGAGATTTGAATATGTGGAAGGAAAAGTGAGTGTAGAT
[10]Copy\of\B108-3...TGTGATAAAAAATGTGAAGGGAAGAGATTTGAATATGTGGAAGGAAAAGTGAGTGTAGAT
[11]Copy\of\B108-3...TGTGATAAAAAATGTGAAGGGAAGAGATTTGAATATGTGGAAGGAAAAGTGAGTGTAGAT
[12]Copy\of\B108-3...TGTGATAAAAAATGTGAAGGGAAGAGATTTGAATATGTGGAAGGAAAAGTGAGTGTAGAT
[13]Copy\of\B108-3...TGTGATAAAAAATGTGAAGGGAAGAGATTTGAATATGTGGAAGGAAAAGTGAGTGTAGAT
[14]Copy\of\B108-3...TGTGATAAAAAATGTGAAGGGAAGAGATTTGAATATGTGGAAGGAAAAGTGAGTGTAGAT
[15]Copy\of\B108-3...TGTGATAAAAAATGTGAAGGGAAGAGATTTGAATATGTGGAAGGAAAAGTGAGTGTAGAT

Genomic_Sequence     TATAAACTTCCAAAAAGACAAGCCCAAAATACACCTAAACGTTATGTCAGATTATTTTGT
[1]Copy\of\B108-35...TATAAATTTTTAAAAAGATAAGTTTAAAATATATTTAAATGTTATGTTAGATTATTTTGT
[2]Copy\of\B108-35...TATAAATTTTTAAAAAGATAAGTTTAAAATATATTTAAATGTTATGTTAGATTATTTTGT
[3]Copy\of\B108-35...TATAAATTTTTAAAAAGATAAGTTTAAAATATATTTAAATGTTATGTTAGATTATTTTGT
[4]Copy\of\B108-35...TATAAATTTTTAAAAAGATAAGTTTAAAATATATTTAAATGTTATGTTAGATTATTTTGT
[5]Copy\of\B108-35...TATAAATTTTTAAAAAGATAAGTTTAAAATATATTTAAATGTTATGTTAGATTATTTTGT
[6]Copy\of\B108-35...TATAAATTTTTAAAAAGATAAGTTTAAAATATATTTAAATGTTATGTTAGATTATTTTGT
[7]Copy\of\B108-35...TATAAATTTTTAAAAAGATAAGTTTAAAATATATTTAAATGTTATGTTAGATTATTTTGT
[8]Copy\of\B108-35...TATAAATTTTTAAAAAGATAAGTTTAAAATATATTTAAATGTTATGTTAGATTATTTTGT
[9]Copy\of\B108-35...TATAAATTTTTAAAAAGATAAGTTTAAAATATATTTAAATGTTATGTTAGATTATTTTGT
[10]Copy\of\B108-3...TATAAATTTTTAAAAAGATAAGTTTAAAATATATTTAAATGTTATGTTAGATTATTTTGT
[11]Copy\of\B108-3...TATAAATTTTTAAAAAGATAAGTTTAAAATATATTTAAATGTTATGTTAGATTATTTTGT
[12]Copy\of\B108-3...TATAAATTTTTAAAAAGATAAGTTTAAAATATATTTAAACGTTATGTTAGATTATTTTGT
[13]Copy\of\B108-3...TATAAATTTTTAAAAAGATAAGTTTAAAATATATTTAAATGTTATGTTAGATTATTTTGT
[14]Copy\of\B108-3...TATAAATTTTTAAAAAGATAAGTTTAAAATATATTTAAATGTTATGTTAGATTATTTTGT
[15]Copy\of\B108-3...TATAAATTTTTAAAAAGATAAGTTTAAAATATATTTAAATGTTATGTTAGATTATTTTGT

Genomic_Sequence     TAAAATCAGTTG
[1]Copy\of\B108-35...TAAAATTAGTTG
[2]Copy\of\B108-35...TAAAATTAGTTG
[3]Copy\of\B108-35...TAAAATTAGTTG
[4]Copy\of\B108-35...TAAAATTAGTTG
[5]Copy\of\B108-35...TAAAATTAGTTG
[6]Copy\of\B108-35...TAAAATTAGTTG
[7]Copy\of\B108-35...TAAAATTAGTTG
[8]Copy\of\B108-35...TAAAATTAGTTG
[9]Copy\of\B108-35...TAAAATTAGTTG
[10]Copy\of\B108-3...TAAAATTAGTTG
[11]Copy\of\B108-3...TAAAATTAGTTG
[12]Copy\of\B108-3...TAAAATTAGTTG
[13]Copy\of\B108-3...TAAAATTAGTTG
[14]Copy\of\B108-3...TAAAATTAGTTG
[15]Copy\of\B108-3...TAAAATTAGTTG
```

Genename MTND4P12

Gene Genomic Sequence (original)
TAGTAGGTTAATAGTGGGGGGTAAGG**CG**AGATTAGTGAGGCTTGCTAGAAGTCATCAAAAGGCTATTAGTGGGAGTAGGGTT
TGAAGTCCTTGAGAGAGAATTATGATG**CG**ACTGTGGGTA**CG**TT**CG**TAGTTTGAGTTTGCTAGGCAGAATAGTAATGAGGATG
TAAGT

a. Bisulfite treatment with Zymo EZ DNA methylation-lightening kit
Genomic_Sequence TAGTAGGTTAATAGTGGGGGGTAAGGCGAGATTAGTGAGGCTTGCTAGAAGTCATCAAAA
[1]Copy\of\B108-35...TAGTAGGTTAATAGTGGGGGGTAAGGTGAGATTAGTGAGGTTTGTTAGAAGTTATTAAAA
[2]Copy\of\B108-35...TAGTAGGTTAATAGTGGGGGGTAAGGTGAGATTAGTGAGGTTTGTTAGAAGTTATTAAAA
[3]Copy\of\B108-35...TAGTAGGTTAATAGTGGGGGGTAAGGTGAGATTAGTGAGGTTTGTTAGAAGTTATTAAAA
[4]Copy\of\B108-35...TAGTAGGTTAATAGTGGGGGGTAAGGTGAGATTAGTGAGGTTTGTTAGAAGTTATTAAAA
[5]Copy\of\B108-35...TAGTAGGTTAATAGTGGGGGGTAAGGTGAGATTAGTGAGGTTTGTTAGAAGTTATTAAAA
[6]Copy\of\B108-35...TAGTAGGTTAATAGTGGGGGGTAAGGTGAGATTAGTGAGGTTTGTTAGAAGTTATTAAAA
[7]Copy\of\B108-35...TAGTAGGTTAATAGTGGGGGGTAAGGTGAGATTAGTGAGGTTTGTTAGAAGTTATTAAAA
[8]Copy\of\B108-35...TAGTAGGTTAATAGTGGGGGGTAAGGTGAGATTAGTGAGGTTTGTTAGAAGTTATTAAAA
[9]Copy\of\B108-35...TAGTAGGTTAATAGTGGGGGGTAAGGTGAGATTAGTGAGGTTTGTTAGAAGTTATTAAAA
[10]Copy\of\B108-3...TAGTAGGTTAATAGTGGGGGGTAAGGTGAGATTAGTGAGGTTTGTTAGAAGTTATTAAAA
[11]Copy\of\B108-3...TAGTAGGTTAATAGTGGGGGGTAAGGTGAGATTAGTGAGGTTTGTTAGAAGTTATTAAAA
[12]Copy\of\B108-3...TAGTAGGTTAATAGTGGGGGGTAAGGTGAGATTAGTGAGGTTTGTTAGAAGTTATTAAAA
[13]Copy\of\B108-3...TAGTAGGTTAATAGTGGGGGGTAAGGTGAGATTAGTGAGGTTTGTTAGAAGTTATTAAAA
[14]Copy\of\B108-3...TAGTAGGTTAATAGTGGGGGGTAAGGTGAGATTAGTGAGGTTTGTTAGAAGTTATTAAAA
[15]Copy\of\B108-3...TAGTAGGTTAATAGTGGGGGGTAAGGTGAGATTAGTGAGGTTTGTTAGAAGTTATTAAAA

Genomic_Sequence GGCTATTAGTGGGAGTAGGGTTTGAAGTCCTTGAGAGAGAATTATGATGCGACTGTGGGT
[1]Copy\of\B108-35...GGTTATTAGTGGGAGTAGGGTTTGAAGTTTTTGAGAGAGAATTATGATGTGATTGTGGGT
[2]Copy\of\B108-35...GGTTATTAGTGGGAGTAGGGTTTGAAGTTTTTGAGAGAGAATTATGATGTGATTGTGGGT
[3]Copy\of\B108-35...GGTTATTAGTGGGAGTAGGGTTTGAAGTTTTTGAGAGAGAATTATGATGTGATTGTGGGT
[4]Copy\of\B108-35...GGTTATTAGTGGGAGTAGGGTTTGAAGTTTTTGAGAGAGAATTATGATGTGATTGTGGGT
[5]Copy\of\B108-35...GGTTATTAGTGGGAGTAGGGTTTGAAGTTTTTGAGAGAGAATTATGATGTGATTGTGGGT
[6]Copy\of\B108-35...GGTTATTAGTGGGAGTAGGGTTTGAAGTTTTTGAGAGAGAATTATGATGTGATTGTGGGT
[7]Copy\of\B108-35...GGTTATTAGTGGGAGTAGGGTTTGAAGTTTTTGAGAGAGAATTATGATGTGATTGTGGGT
[8]Copy\of\B108-35...GGTTATTAGTGGGAGTAGGGTTTGAAGTTTTTGAGAGAGAATTATGATGTGATTGTGGGT
[9]Copy\of\B108-35...GGTTATTAGTGGGAGTAGGGTTTGAAGTTTTTGAGAGAGAATTATGATGTGATTGTGGGT
[10]Copy\of\B108-3...GGTTATTAGTGGGAGTAGGGTTTGAAGTTTTTGAGAGAGAATTATGATGTGATTGTGGGT
[11]Copy\of\B108-3...GGTTATTAGTGGGAGTAGGGTTTGAAGTTTTTGAGAGAGAATTATGATGTGATTGTGGGT
[12]Copy\of\B108-3...GGTTATTAGTGGGAGTAGGGTTTGAAGTTTTTGAGAGAGAATTATGATGTGATTGTGGGT
[13]Copy\of\B108-3...GGTTATTAGTGGGAGTAGGGTTTGAAGTTTTTGAGAGAGAATTATGATGTGATTGTGGGT
[14]Copy\of\B108-3...GGTTATTAGTGGGAGTAGGGTTTGAAGTTTTTGAGAGAGAATTATGATGTGATTGTGGGT
[15]Copy\of\B108-3...GGTTATTAGTGGGAGTAGGGTTTGAAGTTTTTGAGAGAGAATTATGATGTGATTGTGGGT

Genomic_Sequence ACGTTCGTAGTTTGAGTTTGCTAGGCAGAATAGTAATGAGGATGTAAGT
[1]Copy\of\B108-35...ATGTTTGTAGTTTGAGTTTGTTAGGTAGAATAGTAATGAGGATGTAAGT
[2]Copy\of\B108-35...ATGTTTGTAGTTTGAGTTTGTTAGGTAGAATAGTAATGAGGATGTAAGT
[3]Copy\of\B108-35...ATGTTTGTAGTTTGAGTTTGTTAGGTAGAATAGTAATGAGGATGTAAGT
[4]Copy\of\B108-35...ATGTTTGTAGTTTGAGTTTGTTAGGTAGAATAGTAATGAGGATGTAAGT
[5]Copy\of\B108-35...ATGTTTGTAGTTTGAGTTTGTTAGGTAGAATAGTAATGAGGATGTAAGT
[6]Copy\of\B108-35...ATGTTTGTAGTTTGAGTTTGTTAGGTAGAATAGTAATGAGGATGTAAGT
[7]Copy\of\B108-35...ATGTTTGTAGTTTGAGTTTGTTAGGTAGAATAGTAATGAGGATGTAAGT
[8]Copy\of\B108-35...ACGTTTGTAGTTTGAGTTTGTTAGGTAGAATAGTAATGAGGATGTAAGT
[9]Copy\of\B108-35...ATGTTTGTAGTTTGAGTTTGTTAGGTAGAATAGTAATGAGGATGTAAGT
[10]Copy\of\B108-3...ATGTTTGTAGTTTGAGTTTGTTAGGTAGAATAGTAATGAGGATGTAAGT
[11]Copy\of\B108-3...ATGTTTGTAGTTTGAGTTTGTTAGGTAGAATAGTAATGAGGATGTAAGT
[12]Copy\of\B108-3...ATGTTTGTAGTTTGAGTTTGTTAGGTAGAATAGTAATGAGGATGTAAGT
[13]Copy\of\B108-3...ATGTTTGTAGTTTGAGTTTGTTAGGTAGAATAGTAATGAGGATGTAAGT
[14]Copy\of\B108-3...ATGTTTGTAGTTTGAGTTTGTTAGGTAGAATAGTAATGAGGATGTAAGT
[15]Copy\of\B108-3...ATGTTTGTAGTTTGAGTTTGTTAGGTAGAATAGTAATGAGGATGTAAGT

b. Bisulfite treatment with 70℃ for 30 min.

```
Genomic_Sequence      TAGTAGGTTAATAGTGGGGGGTAAGGCGAGATTAGTGAGGCTTGCTAGAAGTCATCAAAA
[1]Copy\of\B108-35...TAGTAGGTTAATAGTGGGGGGTAAGGTGAGATTAGTGAGGTTTGTTAGAAGTTATTAAAA
[2]Copy\of\B108-35...TAGTAGGTTAATAGTGGGGGGTAAGGTGAGATTAGTGAGGTTTGTTAGAAGTTATTAAAA
[3]Copy\of\B108-35...TAGTAGGTTAATAGTGGGGGGTAAGGTGAGATTAGTGAGGTTTGTTAGAAGTTATTAAAA
[4]Copy\of\B108-35...TAGTAGGTTAATAGTGGGGGGTAAGGTGAGATTAGTGAGGTTTGTTAGAAGTTATTAAAA
[5]Copy\of\B108-35...TAGTAGGTTAATAGTGGGGGGTAAGGTGAGATTAGTGAGGTTTGTTAGAAGTTATTAAAA
[6]Copy\of\B108-35...TAGTAGGTTAATAGTGGGGGGTAAGGTGAGATTAGTGAGGTTTGTTAGAAGTTATTAAAA
[7]Copy\of\B108-35...TAGTAGGTTAATAGTGGGGGGTAAGGTGAGATTAGTGAGGTTTGTTAGAAGTTATTAAAA
[8]Copy\of\B108-35...TAGTAGGTTAATAGTGGGGGGTAAGGTGAGATTAGTGAGGTTTGTTAGAAGTTATTAAAA
[9]Copy\of\B108-35...TAGTAGGTTAATAGTGGGGGGTAAGGTGAGATTAGTGAGGTTTGTTAGAAGTTATTAAAA
[10]Copy\of\B108-3...TAGTAGGTTAATAGTGGGGGGTAAGGTGAGATTAGTGAGGTTTGTTAGAAGTTATTAAAA
[11]Copy\of\B108-3...TAGTAGGTTAATAGTGGGGGGTAAGGTGAGATTAGTGAGGTTTGTTAGAAGTTATTAAAA
[12]Copy\of\B108-3...TAGTAGGTTAATAGTGGGGGGTAAGGTGAGATTAGTGAGGTTTGTTAGAAGTTATTAAAA
[13]Copy\of\B108-3...TAGTAGGTTAATAGTGGGGGGTAAGGTGAGATTAGTGAGGTTTGTTAGAAGTTATTAAAA
[14]Copy\of\B108-3...TAGTAGGTTAATAGTGGGGGGTAAGGTGAGATTAGTGAGGTTTGTTAGAAGTTATTAAAA
[15]Copy\of\B108-3...TAGTAGGTTAATAGTGGGGGGTAAGGTGAGATTAGTGAGGTTTGTTAGAAGTTATTAAAA

Genomic_Sequence      GGCTATTAGTGGGAGTAGGGTTTGAAGTCCTTGAGAGAGAATTATGATGCGACTGTGGGT
[1]Copy\of\B108-35...GGTTATTAGTGGGAGTAGGGTTTGAAGTTTTTGAGAGAGAATTATGATGTGATTGTGGGT
[2]Copy\of\B108-35...GGTTATTAGTGGGAGTAGGGTTTGAAGTTTTTGAGAGAGAATTATGATGTGATTGTGGGT
[3]Copy\of\B108-35...GGTTATTAGTGGGAGTAGGGTTTGAAGTTTTTGAGAGAGAATTATGATGTGATTGTGGGT
[4]Copy\of\B108-35...GGTTATTAGTGGGAGTAGGGTTTGAAGTTTTTGAGAGAGAATTATGATGTGATTGTGGGT
[5]Copy\of\B108-35...GGTTATTAGTGGGAGTAGGGTTTGAAGTTTTTGAGAGAGAATTATGATGTGATTGTGGGT
[6]Copy\of\B108-35...GGTTATTAGTGGGAGTAGGGTTTGAAGTTTTTGAGAGAGAATTATGATGTGATTGTGGGT
[7]Copy\of\B108-35...GGTTATTAGTGGGAGTAGGGTTTGAAGTTTTTGAGAGAGAATTATGATGTGATTGTGGGT
[8]Copy\of\B108-35...GGTTATTAGTGGGAGTAGGGTTTGAAGTTTTTGAGAGAGAATTATGATGTGATTGTGGGT
[9]Copy\of\B108-35...GGTTATTAGTGGGAGTAGGGTTTGAAGTTTTTGAGAGAGAATTATGATGTGATTGTGGGT
[10]Copy\of\B108-3...GGTTATTAGTGGGAGTAGGGTTTGAAGTTTTTGAGAGAGAATTATGATGTGATTGTGGGT
[11]Copy\of\B108-3...GGTTATTAGTGGGAGTAGGGTTTGAAGTTTTTGAGAGAGAATTATGATGTGATTGTGGGT
[12]Copy\of\B108-3...GGTTATTAGTGGGAGTAGGGTTTGAAGTTTTTGAGAGAGAATTATGATGTGATTGTGGGT
[13]Copy\of\B108-3...GGTTATTAGTGGGAGTAGGGTTTGAAGTTTTTGAGAGAGAATTATGATGTGATTGTGGGT
[14]Copy\of\B108-3...GGTTATTAGTGGGAGTAGGGTTTGAAGTTTTTGAGAGAGAATTATGATGTGATTGTGGGT
[15]Copy\of\B108-3...GGTTATTAGTGGGAGTAGGGTTTGAAGTTTTTGAGAGAGAATTATGATGTGATTGTGGGT

Genomic_Sequence      ACGTTCGTAGTTTGAGTTTGCTAGGCAGAATAGTAATGAGGATGTAAGT
[1]Copy\of\B108-35...ATGTTTGTAGTTTGAGTTTGTTAGGTAGAATAGTAATGAGGATGTAAGT
[2]Copy\of\B108-35...ATGTTTGTAGTTTGAGTTTGTTAGGTAGAATAGTAATGAGGATGTAAGT
[3]Copy\of\B108-35...ATGTTTGTAGTTTGAGTTTGTTAGGTAGAATAGTAATGAGGATGTAAGT
[4]Copy\of\B108-35...ATGTTTGTAGTTTGAGTTTGTTAGGTAGAATAGTAATGAGGATGTAAGT
[5]Copy\of\B108-35...ATGTTTGTAGTTTGAGTTTGTTAGGTAGAATAGTAATGAGGATGTAAGT
[6]Copy\of\B108-35...ATGTTTGTAGTTTGAGTTTGTTAGGTAGAATAGTAATGAGGATGTAAGT
[7]Copy\of\B108-35...ATGTTTGTAGTTTGAGTTTGTTAGGTAGAATAGTAATGAGGATGTAAGT
[8]Copy\of\B108-35...ATGTTTGTAGTTTGAGTTTGTTAGGTAGAATAGTAATGAGGATGTAAGT
[9]Copy\of\B108-35...ATGTTTGTAGTTTGAGTTTGTTAGGTAGAATAGTAATGAGGATGTAAGT
[10]Copy\of\B108-3...ATGTTTGTAGTTTGAGTTTGTTAGGTAGAATAGTAATGAGGATGTAAGT
[11]Copy\of\B108-3...ATGTTTGTAGTTTGAGTTTGTTAGGTAGAATAGTAATGAGGATGTAAGT
[12]Copy\of\B108-3...ATGTTTGTAGTTTGAGTTTGTTAGGTAGAATAGTAATGAGGATGTAAGT
[13]Copy\of\B108-3...ATGTTTGTAGTTTGAGTTTGTTAGGTAGAATAGTAATGAGGATGTAAGT
[14]Copy\of\B108-3...ATGTTTGTAGTTTGAGTTTGTTAGGTAGAATAGTAATGAGGATGTAAGT
[15]Copy\of\B108-3...ACGTTTGTAGTTTGAGTTTGTTAGGTAGAATAGTAATGAGGATGTAAGT
```

Genename RASSF1A

Gene Genomic Sequence (original)

TTCCATTG**CGCG**GCTCTCCTCAGCTCCTTCC**CG**C**CG**CCCAGTCTGGATCCTGGGGGAGG**CG**CTGAAGT**CG**GGGCC**CG**CCCTG
TGGCCC**CG**CC**CG**GCC**CGCG**CTTGCTAG**CG**CCCAAAGCCAG**CG**AAGCA**CG**GGCCCAAC**CG**GGCCATGT**CG**

a. Bisulfite treatment with Zymo EZ DNA methylation-lightening kit

```
Genomic_Sequence     TTCCATTGCGCGGCTCTCCTCAGCTCCTTCCCGCCGCCCAGTCTGGATCCTGGGGGAGGC
[1]Copy\of\B108-35...TTTTATTGCGCGGTTTTTTTTAGTTTTTTTTTGTCGTTTAGTTTGGATTTTGGGGGAGGC
[2]Copy\of\B108-35...TTTTATTGTGCGGTTTTTTTTAGTTTTTTTTCGTCGTTTAGTTTGGATTTTGGGGGAGGC
[3]Copy\of\B108-35...TTTTATTGCGCGGTTTTTTTTAGTTTTTTTTCGTCGTTTAGTTTGGATTTTGGGGGAGGC
[4]Copy\of\B108-35...TTTTATTGTGCGGTTTTTTTTAGTTTTTTTTTGTTGTTTAGTTTGGATTTTGGGGGAGGT
[5]Copy\of\B108-35...TTTTATTGCGTGGTTTTTTTTAGTTTTTTTTCGTCGTTTAGTTTGGATTTTGGGGGAGGC
[6]Copy\of\B108-35...TTTTATTGCGCGGTTTTTTTTAGTTTTTTTTCGTCGTTTAGTTTGGATTTTGGGGGAGGC
[7]Copy\of\B108-35...TTTTATTGTGCGGTTTTTTTTAGTTTTTTTTCGTCGTTTAGTTTGGATTTTGGGGGAGGT
[8]Copy\of\B108-35...TTTTATTGTGTGGTTTTTTTTAGTTTTTTTTCGTCGTTTAGTTTGGATTTTGGGGGAGGT
[9]Copy\of\B108-35...TTTTATTGCGCGGTTTTTTTTAGTTTTTTTTCGTTGTTTAGTTTGGATTTTGGGGGAGGC
[10]Copy\of\B108-3...TTTTATTGCGTGGTTTTTTTTAGTTTTTTTTCGTCGTTTAGTTTGGATTTTGGGGGAGGC
[11]Copy\of\B108-3...TTTTATTGCGCGGTTTTTTTTAGTTTTTTTTCGTCGTTTAGTTTGGATTTTGGGGGAGGC
[12]Copy\of\B108-3...TTTTATTGTGTGGTTTTTTTTAGTTTTTTTTCGTTGTTTAGTTTGGATTTTGGGGGAGGT
[13]Copy\of\B108-3...TTTTATTGCGCGGTTTTTTTTAGTTTTTTTTCGTCGTTTAGTTTGGATTTTGGGGGAGGC
[14]Copy\of\B108-3...TTTTATTGCGCGGTTTTTTTTAGTTTTTTTTCGTCGTTTAGTTTGGATTTTGGGGGAGGC
[15]Copy\of\B108-3...TTTTATTGCGCGGTTTTTTTTAGTTTTTTTTCGTCGTTTAGTTTGGATTTTGGGGGAGGC

Genomic_Sequence     GCTGAAGTCGGGGCCCGCCCTGTGGCCCCGCCCGGCCCGCGCTTGCTAGCGCCCAAAGCC
[1]Copy\of\B108-35...GTTGAAGTCGGGGTTCGTTTTGTGGTTTCGTTCGGTTCGCGTTTGTTAGCGTTTAAAGTT
[2]Copy\of\B108-35...GTTGAAGTCGGGGTTCGTTTTGTGGTTTCGTTCGGTTCGCGTTTGTTAGTGTTTAAAGTT
[3]Copy\of\B108-35...GTTGAAGTCGGGGTTCGTTTTGTGGTTTCGTTCGGTTCGCGTTTGTTAGCGTTTAAAGTT
[4]Copy\of\B108-35...GTTGAAGTTGGGGTTTGTTTTGTGGTTTTGTTTGGTTCGTGTTTGTTAGTGTTTAAAGTT
[5]Copy\of\B108-35...GTTGAAGTCGGGGTTCGTTTTGTGGTTTCGTTCGGTTCGCGTTTGTTAGCGTTTAAAGTT
[6]Copy\of\B108-35...GTTGAAGTCGGGGTTCGTTTTGTGGTTTCGTTCGGTTCGCGTTTGTTAGCGTTTAAAGTT
[7]Copy\of\B108-35...GTTGAAGTCGGGGTTCGTTTTGTGGTTTCGTTCGGTTCGCGTTTGTTAGCGTTTAAAGTT
[8]Copy\of\B108-35...GTTGAAGTTGGGGTTTGTTTTGTGGTTTCGTTTGGTTTGTGTTTGTTAGTGTTTAAAGTT
[9]Copy\of\B108-35...GTTGAAGTCGGGGTTCGTTTTGTGGTTTCGTTCGGTTCGCGTTTGTTAGCGTTTAAAGTT
[10]Copy\of\B108-3...GTTGAAGTCGGGGTTCGTTTTGTGGTTTCGTTCGGTTCGCGTTTGTTAGCGTTTAAAGTT
[11]Copy\of\B108-3...GTTGAAGTCGGGGTTCGTTTTGTGGTTTCGTTCGGTTCGCGTTTGTTAGCGTTTAAAGTT
[12]Copy\of\B108-3...GTTGAAGTTGGGGTTTGTTTTGTGGTTTCGTTCGGTTTGTGTTTGTTAGTGTTTAAAGTT
[13]Copy\of\B108-3...GTTGAAGTCGGGGTTCGTTTTGTGGTTTCGTTCGGTTCGCGTTTGTTAGCGTTTAAAGTT
[14]Copy\of\B108-3...GTTGAAGTCGGGGTTCGTTTTGTGGTTTCGTTCGGTTCGCGTTTGTTAGCGTTTAAAGTT
[15]Copy\of\B108-3...GTTGAAGTCGGGGTTCGTTTTGTGGTTTCGTTCGGTTCGCGTTTGTTAGCGTTTAAAGTT

Genomic_Sequence     AGCGAAGCACGGGCCCAACCGGGCCATGTCG
[1]Copy\of\B108-35...AGTGAAGTACGGGTTTAATCGGGTTATGTCG
[2]Copy\of\B108-35...AGCGAAGTACGGGTTTAATCGGGTTATGTCG
[3]Copy\of\B108-35...AGCGAAGTACGGGTTTAATCGGGTTATGTCG
[4]Copy\of\B108-35...AGTGAAGTATGGGTTTAATTGGGTTATGTTG
[5]Copy\of\B108-35...AGCGAAGTACGGGTTTAATCGGGTTATGTCG
[6]Copy\of\B108-35...AGCGAAGTACGGGTTTAATCGGGTTATGTCG
[7]Copy\of\B108-35...AGCGAAGTACGGGTTTAATCGGGTTATGTCG
[8]Copy\of\B108-35...AGCGAAGTATGGGTTTAATTGGGTTATGTTG
[9]Copy\of\B108-35...AGCGAAGTACGGGTTTAATCGGGTTATGTCG
[10]Copy\of\B108-3...AGCGAAGTACGGGTTTAATCGGGTTATGTCG
[11]Copy\of\B108-3...AGCGAAGTACGGGTTTAATCGGGTTATGTCG
[12]Copy\of\B108-3...AGCGAAGTATGGGTTTAATTGGGTTATGTTG
[13]Copy\of\B108-3...AGCGAAGTACGGGTTTAATCGGGTTATGTCG
[14]Copy\of\B108-3...AGCGAAGTACGGGTTTAATCGGGTTATGTCG
[15]Copy\of\B108-3...AGCGAAGTACGGGTTTAATCGGGTTATGTCG
```

b. Bisulfite treatment with 70℃ for 30 min

```
Genomic_Sequence      TTCCATTGCGCGGCTCTCCTCAGCTCCTTCCCGCCGCCCAGTCTGGATCCTGGGGGAGGC
[1]Copy\of\B108-35...TTTTATTGCGCGGTTTTTTTTAGTTTTTTTTCGTCGTTTAGTTTGGATTTTGGGGGAGGT
[2]Copy\of\B108-35...TTTTATTGTGTGGTTTTTTTTAGTTTTTTTTTGTTGTTTAGTTTGGATTTTGGGGGAGGC
[3]Copy\of\B108-35...TTTTATTGCGCGGTTTTTTTTAGTTTTTTTTCGTCGTTTAGTTTGGATTTTGGGGGAGGC
[4]Copy\of\B108-35...TTTTATTGCGCGGTTTTTTTTAGTTTTTTTTCGTCGTTTAGTTTGGATTTTGGGGGAGGC
[5]Copy\of\B108-35...TTTTATTGCGCGGTTTTTTTTAGTTTTTTTTCGTCGTTTAGTTTGGATTTTGGGGGAGGC
[6]Copy\of\B108-35...TTTTATTGCGTGGTTTTTTTTAGTTTTTTTTCGTCGTTTAGTTTGGATTTTGGGGGAGGC
[7]Copy\of\B108-35...TTTTATTGCGCGGTTTTTTTTAGTTTTTTTTCGTCGTTTAGTTTGGATTTTGGGGGAGGC
[8]Copy\of\B108-35...TTTTATTGTGCGGTTTTTTTTAGTTTTTTTTTGTTGTTTAGTTTGGATTTTGGGGGAGGT
[9]Copy\of\B108-35...TTTTATTGCGCGGTTTTTTTTAGTTTTTTTTCGTCGTTTAGTTTGGATTTTGGGGGAGGC
[10]Copy\of\B108-3...TTTTATTGCGCGGTTTTTTTTAGTTTTTTTTCGTCGTTTAGTTTGGATTTTGGGGGAGGC
[11]Copy\of\B108-3...TTTTATTGCGCGGTTTTTTTTAGTTTTTTTTCGTCGTTTAGTTTGGATTTTGGGGGAGGC
[13]Copy\of\B108-3...TTTTATTGTGCGGTTTTTTTTAGTTTTTTTTTGTTGTTTAGTTTGGATTTTGGGGGAGGT
[14]Copy\of\B108-3...TTTTATTGCGCGGTTTTTTTTAGTTTTTTTTCGTCGTTTAGTTTGGATTTTGGGGGAGGC
[15]Copy\of\B108-3...TTTTATTGCGCGGTTTTTTTTAGTTTTTTTTCGTCGTTTAGTTTGGATTTTGGGGGAGGC

Genomic_Sequence      GCTGAAGTCGGGGCCCGCCCTGTGGCCCCGCCCGGCCCGCGCTTGCTAGCGCCCAAAGCC
[1]Copy\of\B108-35...GTTGAAGTCGGGGTTCGTTTTGTGGTTTCGTTCGGTTCGCGTTTGTTAGCGTTTAAAGTT
[2]Copy\of\B108-35...GTTGAAGTTGGGGTTCGTTTTGTGGTTTTGTTTGGTTCGTGTTTGTTAGTGTTTAAAGTT
[3]Copy\of\B108-35...GTTGAAGTCGGGGTTCGTTTTGTGGTTTCGTTCGGTTCGCGTTTGTTAGCGTTTAAAGTT
[4]Copy\of\B108-35...GTTGAAGTCGGGGTTCGTTTTGTGGTTTCGTTCGGTTCGCGTTTGTTAGCGTTTAAAGTT
[5]Copy\of\B108-35...GTTGAAGTCGGGGTTCGTTTTGTGGTTTCGTTCGGTTCGTGTTTGTTAGCGTTTAAAGTT
[6]Copy\of\B108-35...GTTGAAGTCGGGGTTTGTTTTGTGGTTTCGTTCGGTTCGCGTTTGTTAGCGTTTAAAGTT
[7]Copy\of\B108-35...GTTGAAGTCGGGGTTCGTTTTGTGGTTTCGTTTGGTTCGCGTTTGTTAGCGTTTAAAGTT
[8]Copy\of\B108-35...GTTGAAGTTGGGGTTTGTTTTGTGGTTTTGTTTGGTTCGTGTTTGTTAGTGTTTAAAGTT
[9]Copy\of\B108-35...GTTGAAGTCGGGGTTCGTTTTGTGGTTTCGTTCGGTTCGCGTTTGTTAGCGTTTAAAGTT
[10]Copy\of\B108-3...GTTGAAGTCGGGGTTCGTTTTGTGGTTTCGTTCGGTTCGCGTTTGTTAGCGTTTAAAGTT
[11]Copy\of\B108-3...GTTGAAGTCGGGGTTCGTTTTGTGGTTTCGTTCGGTTCGCGTTTGTTAGCGTTTAAAGTT
[12]Copy\of\B108-3...GTTGAAGTCGGGGTTCGTTTTGTGGTTTCGTTCGGTTCGCGTTTGTTAGCGTTTAAAGTT
[13]Copy\of\B108-3...GTTGAAGTTGGGGTTTGTTTTGTGGTTTTGTTTGGTTCGTGTTTGTTAGTGTTTAAAGTT
[14]Copy\of\B108-3...GTTGAAGTCGGGGTTCGTTTTGTGGTTTCGTTCGGTTCGCGTTTGTTAGCGTTTAAAGTT
[15]Copy\of\B108-3...GTTGAAGTCGGGGTTCGTTTTGTGGTTTCGTTCGGTTCGCGTTTGTTAGCGTTTAAAGTT

Genomic_Sequence      AGCGAAGCACGGGCCCAACCGGGCCATGTCG
[1]Copy\of\B108-35...AGCGAAGTACGGGTTTAATCGGGTTATGTCG
[2]Copy\of\B108-35...AGTGAAGTATGGGTTTAATTGGGTTATGTTG
[3]Copy\of\B108-35...AGCGAAGTACGGGTTTAATCGGGTTATGTCG
[4]Copy\of\B108-35...AGCGAAGTACGGGTTTAATCGGGTTATGTCG
[5]Copy\of\B108-35...AGCGAAGTACGGGTTTAATTGGGTTATGTCG
[6]Copy\of\B108-35...AGCGAAGTACGGGTTTAATCGGGTTATGTCG
[7]Copy\of\B108-35...AGCGAAGTACGGGTTTAATCGGGTTATGTCG
[8]Copy\of\B108-35...AGTGAAGTATGGGTTTAATTGGGTTATGTTG
[9]Copy\of\B108-35...AGCGAAGTACGGGTTTAATCGGGTTATGTCG
[10]Copy\of\B108-3...AGTGAAGTACGGGTTTAATCGGGTTATGTCG
[11]Copy\of\B108-3...AGCGAAGTACGGGTTTAATCGGGTTATGTCG
[12]Copy\of\B108-3...AGCGAAGTACGGGTTTAATCGGGTTATGTCG
[13]Copy\of\B108-3...AGTGAAGTATGGGTTTAATTGGGTTATGTTG
[14]Copy\of\B108-3...AGCGAAGTACGGGTTTAATCGGGTTATGTCG
[15]Copy\of\B108-3...AGCGAAGTACGGGTTTAATCGGGTTATGTCG
```
